# A deep learning framework for automated quantification of peripheral nerve lesions in MR neurography

**DOI:** 10.1186/s41747-026-00786-z

**Published:** 2026-09-08

**Authors:** Nedim C. Beste, Clara Raudonat, Melissa Fesselier, Sebastian Hartmann, Daniel Sommer, Johann M. E. Jende, Jonathan Kottlors, Sabine Heiland, Hagen Meredig, Martin Bendszus, Christoph M. Mooshage

**Affiliations:** 1 Institute for Diagnostic and Interventional Radiology, Faculty of Medicine, University of Cologne, Cologne, Germany; 2 Department of Neuroradiology, Heidelberg University Hospital, Heidelberg, Germany; 3 German Cancer Research Center (DKFZ), Heidelberg, Germany

**Keywords:** Artificial intelligence, Magnetic resonance imaging, Neural networks (Computer), Periphereal nervous system dieseases, Sciatic neuropathy

## Abstract

**Objective:**

Magnetic resonance neurography (MRN) enables high-resolution visualization of peripheral nerves, yet quantitative analysis of intraneural lesion burden remains limited by manual, observer-dependent workflows. We propose a deep learning-based framework for automated segmentation and quantitative characterization of peripheral nerve lesions in MRN.

**Materials and methods:**

In this retrospective study (*n* = 178), standardized axial fat-suppressed T2-weighted 3-T MRN scans of the distal thigh were analyzed. Sciatic nerves and intraneural lesions were manually annotated to train a modified nnU-Net v2 architecture for 2D and 3D segmentation. Model performance was evaluated using Dice and surface Dice metrics. Generalizability was assessed across two independent test datasets. Imaging-derived lesion burden metrics were further related to electrophysiological parameters using age-adjusted partial correlation analyses.

**Results:**

Automated nerve segmentation achieved a median Dice coefficient of 0.97. Lesion segmentation yielded surface Dice values of 0.64–0.77 across training and independent test sets, demonstrating stable generalization in a small-structure segmentation setting. Quantitative lesion burden metrics were consistent across cohorts and showed significant associations with electrophysiological measures of nerve function. For example, automated total lesion load correlated significantly with tibial nerve conduction velocity (*r* = -0.62, *p* < 0.001) and tibial compound motor action potential (*r* = -0.568, *p* 0.002) in age-adjusted partial correlation analyses.

**Conclusion:**

This work introduces a reproducible deep learning-based framework for automated segmentation and quantitative assessment of peripheral nerve lesions in MR neurography. The approach enables standardized volumetric characterization of small intraneural abnormalities and provides a basis for scalable, quantitative peripheral nerve imaging.

**Key Points:**

- ***Question***: AI automatically detects and measures nerve damage in MRI scans.
- ***Findings***: Method provides a fast, consistent alternative to manual image analysis.
- ***Relevance statement***: Automated MRI-based nerve lesion quantification enables objective, reproducible assessment of peripheral neuropathies, potentially improving diagnostic accuracy, monitoring disease progression, and supporting standardized, scalable evaluation in both clinical practice and research.

**Graphical Abstract:**

**Figure:**
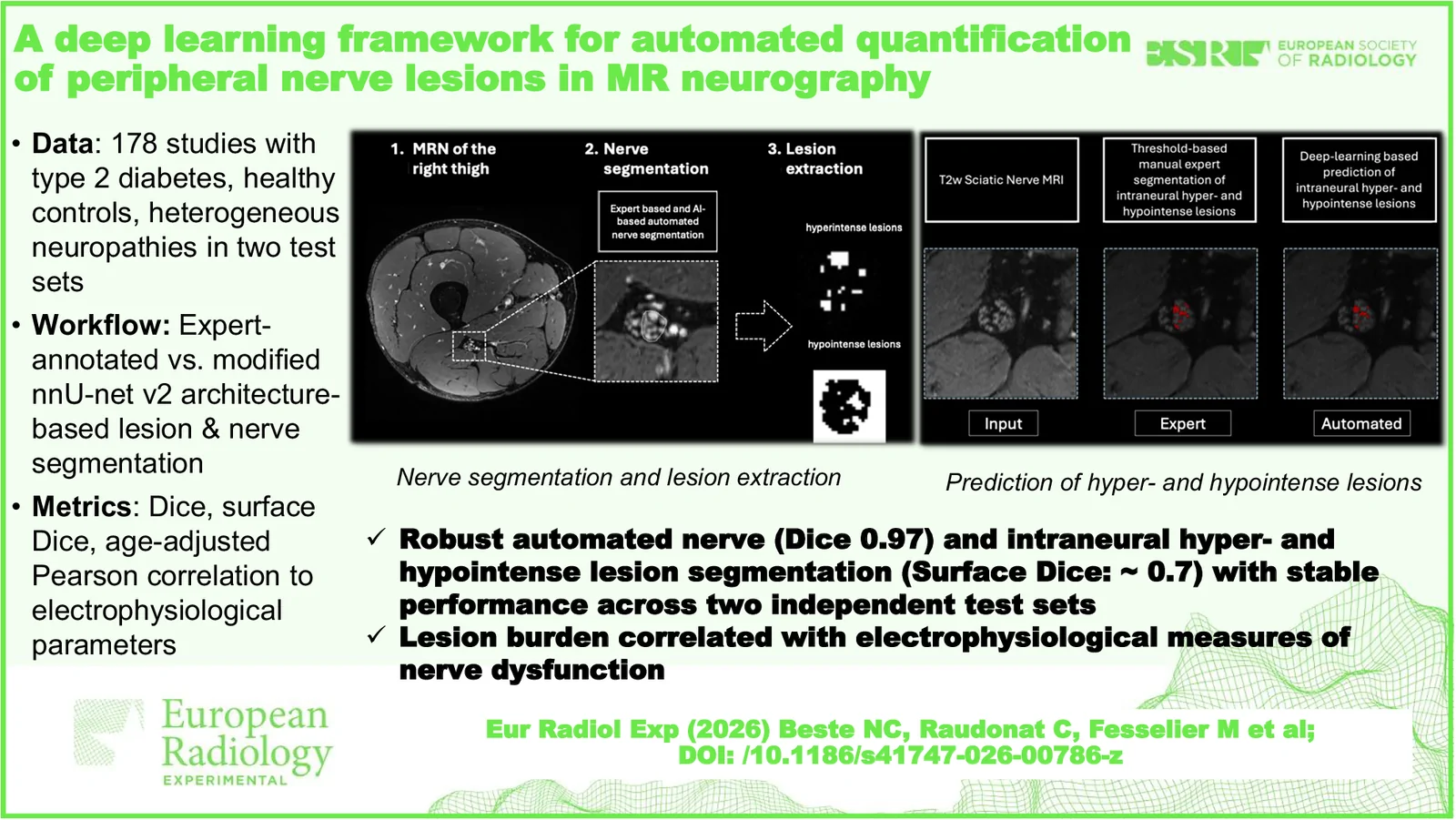

## Introduction

Magnetic resonance neurography (MRN) is a specialized magnetic resonance imaging (MRI) technique for high-resolution visualization of peripheral nerves and plexuses. T2-weighted (T2w), fat-suppressed sequences are the mainstay of MRN, which highlight nerves against surrounding tissues and allow for morphological abnormalities of peripheral nerves [1, 2].

Clinically, MRN aids in diagnosing post-traumatic, inflammatory, hereditary, and metabolic neuropathies, as well as peripheral nerve tumors, especially when clinical and paraclinical examinations cannot precisely localize nerve lesions or assess their extent and distribution. These limitations are most pronounced for proximal nerve segments such as the sciatic nerve, lumbosacral plexus, and spinal nerve roots, which are poorly assessable by standard electrophysiological nerve studies [3, 4].

Beyond its clinical utility, MRN has been applied to provide new insights into the pathophysiology of diffuse polyneuropathies in which T2w hyperintense and hypointense lesions of the sciatic nerve have emerged as key imaging features of structural nerve damage [5]. However, quantitative and reproducible characterization of intraneural abnormalities remains an unresolved challenge in computational neuroimaging, particularly for small-scale structures. Radiologists must distinguish true intraneural signal abnormalities from artifacts, partial-volume effects, intraneural vessels, and adjacent fat, often by tracking nerves across long segments in multiple planes [3, 4]. Moreover, manual lesion annotation is labor-intensive and prone to inter-observer variability. These challenges motivate the need for automated, objective methods to evaluate nerve lesions.

Deep learning offers a potential solution. Convolutional neural networks (CNNs) have become standard in medical imaging segmentation, with U-Net-based architectures achieving robust performance even on limited datasets [6–8]. Beste et al recently developed an nnU-Net model for automatic sciatic nerve segmentation in MRN, achieving high Dice similarity coefficients in cross-validation and on an external test set [9]. This demonstrates the technical feasibility of automated nerve segmentation and lays the groundwork for quantitative MRN analysis. However, prior work has focused on nerve segmentation rather than intraneural lesion segmentation. Here, we present a CNN-based automated segmentation pipeline of hyperintense and hypointense intraneural lesions in the sciatic nerve on MR neurography, providing an objective, reproducible tool for nerve lesion assessment and introducing quantitative and objective MRN biomarkers. The primary aim of this study was the technical validation of a deep learning-based segmentation framework for intraneural lesions of the sciatic nerve in MRN. As a secondary, exploratory objective, we assessed the biological plausibility of automatically derived lesion burden metrics through age-adjusted partial correlation analyses with electrophysiological nerve function parameters. Together, these objectives address the methodological and translational prerequisites for future imaging biomarker development, rather than establishing immediate clinical applicability.

## Materials and methods

### Ethics

The study was approved by the local Ethics Committee of Heidelberg University (local ethics numbers: S398-2012; S-383/2016; S-203/2023). All procedures were performed in accordance with the ethical standards of the institutional research committee and the Declaration of Helsinki and its later amendments. Written informed consent was obtained from all participants.

### Image acquisition

MRN of the thigh was performed using a standardized axial fat-suppressed T2w turbo spin-echo (TSE) sequence. MRI examinations were conducted on clinical 3.0-T whole-body systems (Siemens Healthineers) equipped with a 15-channel transmit/receive knee array coil. Imaging was performed at the Department of Neuroradiology, University Hospital Heidelberg (Magnetom Trio and Prisma, Siemens Healthineers; Training Dataset and Test Set 2) and at the MRI Facility Neuer Wall (Magnetom Skyra, Siemens Healthineers; Test Set 2). A comprehensive overview of scanner models and sequence parameters derived from the DICOM headers is provided in Table 1.

**Table 1:** Overview of sequence parameters

Field strength (T)	3.00 (3.00–3.00)	3.00 (3.00–3.00)	3.00 (3.00–3.00)
TR (ms)	6,960 (6,960–8,150)	6,960 (6,960–6,960)	5,970 (5,970–5,970)
TE (ms)	54 (54–54)	54 (54–54)	55 (55–55)
Flip angle (°)	150 (150–150)	150 (150–150)	150 (150–150)
Echo train length	13 (13–13)	13 (13–13)	13 (13–13)
Slice thickness (mm)	3.50 (3.50–3.50)	3.50 (3.50–3.50)	4.00 (4.00–4.00)
Spacing between slices (mm)	3.85 (3.85–3.85)	3.85 (3.85–3.85)	4.80 (4.80–4.80)
Slice gap (mm)	0.35 (0.35–0.35)	0.35 (0.35–0.35)	0.80 (0.80–0.80)
In-plane resolution (mm)	0.31 (0.31–0.31)	0.31 (0.31–0.31)	0.31 (0.31–0.31)
Matrix	512 × 512	512 × 512	512 × 512

Data presented as median (range)

*ms* Milliseconds, *T* Tesla

### Patient cohort

Subjects were included in this retrospective analysis and identified from the institutional prospectively collected MRN database of the Department of Neuroradiology at the University Hospital Heidelberg. The training dataset comprised healthy control participants and patients with diabetic neuropathy related to type 2 diabetes mellitus presenting with neuropathic intraneural lesions. Two independent test datasets were assembled to evaluate model generalizability. Test dataset 1 included a heterogeneous cohort of patients with diabetic neuropathy, healthy control participants, patients with peripheral nerve schwannomas (Schwannomatosis and Neurofibromatosis Type 2), patients with Fabry disease, and amyotrophic lateral sclerosis [10]. Test dataset 2 comprised patients with diabetic neuropathy in patients with type 1 diabetes, healthy controls, and patients with uremic neuropathy for whom nerve conduction study results were available.

### Annotation procedure

Nerve annotation and lesion segmentation were performed independently by two readers:

- one board-certified radiologist with > 5 years of MRN experience and
- one radiology resident with > 3 years of MRN experience.

Manual lesion annotation was performed in ImageJ using a semi-automated threshold-based workflow. First, the sciatic nerve was manually delineated on each axial T2w slice to restrict subsequent lesion segmentation to the intraneural compartment. Within this contour, T2w hyperintense and hypointense lesion masks were generated using ImageJ version 1.53k via reader-guided intensity thresholding with the “Threshold” function, defining cut-off gray-scale values on 8-bit images. Threshold values were determined independently by each reader on a per-slice basis; no fixed global threshold was applied across subjects or image slices to account for interindividual differences in signal intensity, coil sensitivity, and regional image contrast. For hypointense lesions, the surrounding subcutaneous fat tissue served as the anatomical reference signal. Hypointense lesions were therefore defined as focal intraneural areas with signal characteristics approaching those of adjacent subcutaneous fat. For hyperintense lesions, readers used visually normal nerve fascicles and adjacent healthy muscle tissue as internal references, with hyperintense lesions defined as focal intraneural regions exceeding the expected background signal of normal nerve parenchyma and surrounding muscle. After automated mask generation, each reader manually reviewed and corrected the masks to exclude false-positive segmentations wherever possible. Signal alterations confined to a single slice, clearly linear or tubular structures consistent with intraneural or adjacent vascular structures, and regions discordant with the morphology or signal pattern of the nerve on adjacent slices were excluded when identifiable. Interrater reliability between the two predefined raters was assessed using a two-way mixed-effects model with absolute agreement for single measurements (ICC(3,1), as described by Shrout and Fleiss [11]).

### Semi-automated lesion extraction

Lesions were further processed using a custom-made semi-automated workflow (developed by N.C.B.).

**Fig. 1:**
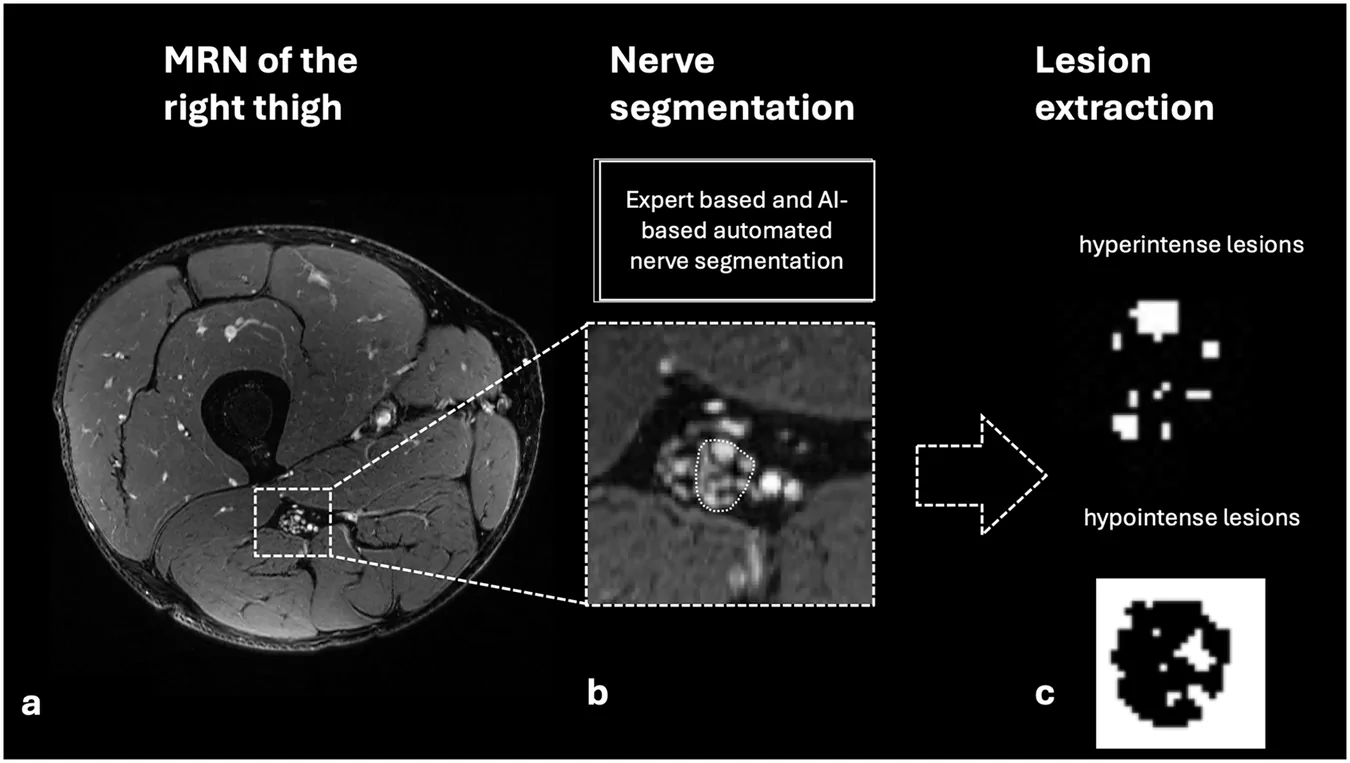
Lesion segmentation. **a** Axial, fat-saturated T2w MRN of the distal right thigh with the sciatic nerve marked with a square. **b** Enlarged image of the sciatic nerve with manual delineation of the tibial compartment of the sciatic nerve. **c** Binary lesion maps of T2w-hyper- and T2w-hypointense nerve lesions

The corresponding workflow was implemented in Python using nibabel, combined with an ImageJ macro for automated slice-wise thresholding. For each slice, the workflow applied automated thresholding, exported the threshold mask, and binarized the lesion regions. These binary lesion maps were used for model training and quantitative analysis. For the evaluation in the test datasets, lesion extraction and subsequent segmentation were performed using two different sources for nerve region definition. In a first approach, lesions were extracted based on expert-annotated sciatic nerve masks. In a second approach, lesions were extracted based on automatically generated nerve masks obtained from the nerve segmentation model. This resulted in two separate lesion mask sets per examination, enabling a direct comparison of lesion segmentation performance depending on whether expert-defined or automatically generated nerve masks were used as the anatomical constraint. Both lesion mask sets were processed identically in all subsequent analyses (Fig. 1).

### Model training

A modified nnU-Net v2 framework was used to train both 2D and 3D full-resolution models.

All models were trained for 1,000 epochs, following the standard nnU-Net preprocessing, augmentation, and postprocessing pipeline. Modifications were introduced to improve segmentation of very small intraneural lesions, including class weighting [0.05, 2.0, 2.0] for background, hyperintense and hypointense lesions to mitigate class imbalance, a combined Focal and Soft Dice loss\(\(α = 0.5\), γ = 3.0) to emphasize hard-to-classify voxels, and a Top‑k voxel mining strategy focusing the loss on the 10% highest-error voxels in each batch. Except for these adaptations, the default nnU-Net v2 configuration was preserved to ensure methodological comparability. To support reproducibility, we report the following implementation details. Two separate nnU-Net v2 models were trained: a nerve segmentation model trained on the full MRI field of view (median image size 20 × 510 × 510 voxels at isotropic 1.0 mm spacing) and a lesion segmentation model trained on images cropped to the nerve region of interest (median size 20 × 39 × 51 voxels). For the nerve segmentation model, the 2D configuration used a PlainConvUNet with 8 encoder stages and feature maps from 32 to 512, patch size 512 × 512 and batch size 12; the 3D full-resolution configuration used 7 encoder stages with feature maps from 32 to 320, patch size 20 × 256 × 256 and batch size 4. For the lesion segmentation model, the 2D configuration used 4 encoder stages with feature maps from 32 to 256, patch size 40 × 56 and batch size 79; the 3D configuration used patch size 20 × 40 × 56 and batch size 4. Both models used the Adam optimizer with an initial learning rate of 0.01 and polynomial decay, Instance Normalization, and Leaky ReLU activation. Nerve segmentation used global *Z*-score normalization; lesion segmentation used foreground-masked *Z*-score normalization after cropping to the nerve region of interest. Model performance was evaluated using 5-fold cross-validation on the training dataset. Inference used test-time augmentation with 8 mirroring axes. Post-processing consisted of removal of connected lesion components below 10 mm³. Class weights of 0.05 for background and 2.0 for each lesion class were chosen to counteract the severe foreground-background class imbalance. The Focal loss with γ = 3.0 increased the training signal on hard-to-classify boundary voxels, and the Top-k strategy with *k* = 10% directed loss computation to the highest-error voxels per batch.

### Volume analysis

Nerve and lesion volumes were quantified in the test datasets using voxel-based volume measurements derived from the DICOM header files. The total volume of nerve and intraneural lesion tissue was calculated by counting the respective voxels and converting these counts into physical volumes based on the underlying image resolution. Volumes are reported in cubic millimeters.

For each subject, a lesion-to-nerve volume ratio was calculated to provide a normalized measure of lesion burden. Nerve and lesion volumes as well as lesion-to-nerve volume ratios were subsequently correlated with available clinical parameters, including nerve conduction study results and clinical measures of neuropathic symptom severity, using Pearson correlation analysis.

### Statistical analysis

Statistical analyses were performed using RStudio [version 2022.12.0 + 353] and Python [version 3.9.7].

A *p*-value < 0.05 was considered statistically significant. Normality of continuous variables was assessed using the Shapiro–Wilk test. The primary performance metric for nerve segmentation was the median Dice similarity coefficient. For lesion segmentation, the primary metric was the median surface Dice, chosen because small 2-D lesions exhibit substantial sensitivity to voxel-level Dice fluctuations and surface-based metrics more robustly represent boundary accuracy in small, focal structures [12]. Median values were chosen because of non-normally distributed data.

Secondary performance metrics included region-based overlap measures, classification-based metrics, and boundary-based distance measures. Specifically, secondary metrics comprised the intersection-over-union (IoU), sensitivity (recall) and the positive predictive value (PPV). We note that the presented metrics should be interpreted in the context of the small individual lesion sizes characteristic of this task, as Dice-based metrics are known to be strongly size-dependent for focal structures [13, 14]. Lesion volume per subject, as reported in the volume analysis section, serves as the primary measure of lesion burden rather than individual lesion component counts.

Partial correlation analysis was conducted to correlate the absolute lesion loads of T2w-hyper- and hypointense lesions as well as overall lesion load with the obtained electrophysiological data in Test Set 2. These analyses were predefined as exploratory secondary endpoints. Given the exploratory nature, the relatively small sample size of Test Set 2 (*n* = 31) and the multiple parameters tested across three lesion types and several electrophysiological measures, no correction for multiple comparisons was applied. All correlation results should therefore be interpreted as hypothesis-generating and not as confirmatory evidence.

## Results

### Patient demographics

In total, 178 subjects were included in the analysis. The training dataset comprised 91 subjects, while test dataset 1 and test dataset 2 included 56 and 31 subjects, respectively. A detailed overview of demographic characteristics and diagnostic composition of the study population is provided in Table 2.

**Table 2:** Study cohort characteristics and lesion segmentation performance

Cohort	Diagnosis	*n*	Age (years) mean ± SD (range)	Sex m/f	Laterality R/L	Role
A—cohort demographics						
Training	Type 2 diabetic polyneuropathy	56/55¹	61.8 ± 8.8 (30–75)	24/32	n/a	Training
	Healthy controls	35/34¹	53.9 ± 12.3 (30–77)	25/10	n/a	
	Total¹	91/89¹	58.8 ± 10.9 (30–77)	49/42	n/a	
Test set 1	Schwannomatosis	14	52.5 ± 15.6 (24–82)	7/7	6 R/8 L	External test
	Healthy controls	12	39.8 ± 12.2 (21–63)	6/6	4 R/8 L	
	Amyotrophic lateral sclerosis	11	59.4 ± 9.3 (48–76)	5/6	6 R/5 L	
	Neurofibromatosis type 2	10	45.1 ± 17.5 (22–68)	3/7	7 R/3 L	
	Fabry disease	9	47.0 ± 13.8 (22–71)	4/5	2 R/7 L	
	Total²	56	48.9 ± 15.1 (21–82)	25/31	25 R/31 L	
Test set 2	Type 1 diabetic polyneuropathy	11	46.9 ± 20.1 (18–70)	4/7	11 R/0 L	External test
	Healthy controls	10	53.7 ± 18.0 (27–70)	7/3	10 R/0 L	
	Uremic polyneuropathy	10	63.2 ± 18.7 (28–86)	3/7	10 R/0 L	
	Total³	31	54.4 ± 19.6 (18–86)	14/17	31 R/0 L	
B—lesion segmentation performance in test set (median, 95% bootstrap CI)						
**Test Set**	**Pipeline**	**n**	**Dice (95% CI)**	**Sensitivity (95% CI)**	**Surface Dice (95% CI)**	**PPV (95% CI)**
Test Set 1	Expert nerve mask – hyperintense	55	0.694 (0.623–0.745)	0.601 (0.502–0.669)	0.871 (0.844–0.912)	0.949 (0.927–0.981)
	*Hypointense*	55	0.657 (0.595–0.716)	0.522 (0.444–0.654)	0.855 (0.830–0.901)	1.000 (0.994–1.000)
Test Set 1	AI nerve mask – hyperintense	55	0.598 (0.535–0.659)	0.489 (0.407–0.608)	0.832 (0.771–0.856)	0.892 (0.852–0.935)
	*Hypointense*	55	0.497 (0.426–0.550)	0.394 (0.330–0.455)	0.748 (0.637–0.788)	0.805 (0.734–0.883)
Test Set 2	Expert nerve mask – hyperintense	30	0.658 (0.606–0.692)	0.528 (0.494–0.579)	0.858 (0.830–0.891)	0.948 (0.908–0.979)
	*Hypointense*	30	0.617 (0.549–0.676)	0.456 (0.386–0.530)	0.807 (0.792–0.868)	1.000 (0.979–1.000)
Test Set 2	AI nerve mask – hyperintense	30	0.587 (0.496–0.627)	0.442 (0.375–0.496)	0.800 (0.741–0.843)	0.916 (0.867–0.956)
	*Hypointense*	30	0.437 (0.370–0.491)	0.321 (0.276–0.380)	0.665 (0.639–0.710)	0.904 (0.841–0.971)

95% bootstrap CIs computed using 1,000 iterations (seed 42). Expert Nerve Mask: lesion segmentation using manually delineated nerve contour. AI Nerve Mask: fully automated end-to-end pipeline. Nerve segmentation performance (median Dice 0.97, surface Dice 1.00) is reported in the main text

*ALS* Amyotrophic lateral sclerosis, *CI* Confidence interval, *f* Female, *HC* Healthy control, *m* Male, *NF2* Neurofibromatosis type 2, *PPV* Positive predictive value, *T1D* Type 1 diabetic polyneuropathy, *T2D* Type 2 diabetic polyneuropathy, *UPNP* Uremic polyneuropathy

¹ Training: 91 subjects for nerve segmentation model; 89 for lesion segmentation model after exclusion of two subjects with annotation quality issues. Patient-level separation between training and test sets was strictly enforced. Laterality was not systematically recorded for the training cohort. As the model was trained on nerve segments cropped to a standardized region of interest without anatomical orientation encoding, laterality does not affect model training or performance evaluation

² Test set 1: one subject excluded from performance analysis due to motion artefacts (demographic *n* = 56, performance *n* = 55)

³ Test set 2: one subject excluded from performance analysis due to a missing prediction file (demographic *n* = 31, performance *n* = 30)

### Nerve and lesion segmentation

Automated sciatic nerve segmentation demonstrated robust performance across datasets, achieving a median Dice coefficient of 0.97 and a median surface Dice of 1.00 in the test datasets (Table 2). Lesion segmentation performance was stable across training and independent test sets, with median surface Dice values ranging from 0.687 to 0.771 and Dice coefficients from 0.613 to 0.731 across lesion types. Performance remained comparable across both external test cohorts, supporting good generalizability (Table 2).

### Interrater agreement

Interrater agreement was assessed in the training dataset using a two-way mixed-effects model for absolute agreement to assess the intraclass correlation coefficient (ICC(3,1)) between two independent readers (CM and CR). The analysis demonstrated excellent agreement, with an ICC of 0.93 (95% CI: 0.88–0.96; *p* < 0.001), indicating high consistency and reproducibility of manual nerve and lesion annotations between readers.

### Influence of nerve mask source

In the test sets, lesion segmentation results were evaluated using two different nerve mask sources: expert-annotated sciatic nerve masks and automatically generated nerve masks derived from the nerve segmentation model. Lesions were segmented once based on the expert-defined nerve masks and once based on the automatically generated nerve masks. While performance metrics were slightly higher when expert nerve masks were used, lesion segmentation based on automatically generated nerve masks achieved comparable median surface Dice and Dice similarity coefficients, indicating robustness of the lesion segmentation pipeline with respect to upstream nerve segmentation variability. A detailed comparison is provided in Table 2.

### Lesion volume analysis in the test sets

Lesion volume characteristics were analyzed separately for the two independent test datasets.

In test set 1, the median hyperintense lesion volume per subject was 73.7 mm³, whereas the median hypointense lesion volume was 89.1 mm³, resulting in a median total lesion volume of 179.9 mm³. When normalized to sciatic nerve volume, median hyperintense and hypointense lesion fractions were 0.063 and 0.076, respectively, corresponding to a total lesion-to-nerve volume ratio of 0.143.

In test set 2, lesion volumes were slightly higher. Median hyperintense and hypointense lesion volumes per subject were 94.7 mm³ and 121.9 mm³, respectively, yielding a median total lesion volume of 223.3 mm³. Relative to nerve volume, hyperintense lesions accounted for a median fraction of 0.067, hypointense lesions for 0.077, and the combined lesion burden corresponded to a total lesion-to-nerve volume ratio of 0.154.

Overall, lesion volume distributions were comparable across both test datasets, with consistently slightly higher hypointense than hyperintense lesion burden and stable normalized lesion-to-nerve volume ratios.

### Correlation analysis of nerve lesion volumes with neurophysiological data in test set 2

In age-adjusted partial correlation analyses, tibial nerve parameters and overall lesion burden showed the most consistent associations with electrophysiological impairment. Manually derived T2w-hyperintense lesion volume and total lesion load were associated with multiple electrophysiological measures, whereas T2w-hypointense lesion volume showed only limited associations. Fully automated lesion quantification demonstrated similarly robust, partly stronger associations, with total lesion load showing the most consistent relationships across electrophysiological parameters (Table 3).

**Table 3:** Results of the partial correlation analysis between the absolute volumes of T2w-hyperintense, T2w-hypointense, and total lesion load in the tibial portion of the sciatic nerve, derived from fully manual and fully automated nerve segmentation and lesion segmentation with electrophysiological parameters adjusted for age

Ground truth	*r*	*p*-value	Fully automated nerve lesion segmentation	*r*	*p*-value
**T2w-hyperintense lesions**			**T2w-hyperintense lesions**		
NCV tibial nerve	-0.575	0.002	NCV tibial nerve	-0.548	0.004
CMAP tibial nerve	-0.519	0.007	CMAP tibial nerve	-0.432	0.028
DML tibial nerve	0.154	0.453	DML tibial nerve	0.292	0.148
NCV peroneal nerve	0.336	0.100	NCV peroneal nerve	0.536	0.006
CMAP peroneal nerve	-0.430	0.100	CMAP peroneal nerve	0.480	0.015
DML peroneal nerve	0.183	0.381	DML peroneal nerve	0.279	0.177
NCV sural nerve	-0.475	0.029	NCV sural nerve	-0.074	0.751
SNAP sural nerve	-0.650	< 0.001	SNAP sural nerve	-0.465	0.025
**T2w-hypointense lesions**			**T2w-hypointense lesions**		
NCV tibial nerve	-0.320	0.111	NCV tibial nerve	-0.497	0.010
CMAP tibial nerve	-0.192	0.347	CMAP tibial nerve	-0.509	0.008
DML tibial nerve	0.159	0.439	DML tibial nerve	0.301	0.136
NCV peroneal nerve	-0.240	0.249	NCV peroneal nerve	-0.675	< 0.001
CMAP peroneal nerve	-0.246	0.235	CMAP peroneal nerve	-0.577	0.003
DML peroneal nerve	0.113	0.592	DML peroneal nerve	0.452	0.023
NCV sural nerve	-0.521	0.015	NCV sural nerve	-0.302	0.183
SNAP sural nerve	-0.274	0.205	SNAP sural nerve	-0.344	0.108
**Overall lesion load**			**Overall lesion load**		
NCV tibial nerve	-0.652	< 0.001	NCV tibial nerve	-0.626	< 0.001
CMAP tibial nerve	-0.526	0.006	CMAP tibial nerve	-0.568	0.002
DML tibial nerve	0.223	0.274	DML tibial nerve	0.357	0.074
NCV peroneal nerve	-0.411	0.041	NCV peroneal nerve	-0.726	< 0.001
CMAP peroneal nerve	-0.487	0.014	CMAP peroneal nerve	-0.633	< 0.001
DML peroneal nerve	0.213	0.307	DML peroneal nerve	0.440	0.028
NCV sural nerve	-0.586	0.005	NCV sural nerve	-0.249	0.277
SNAP sural nerve	-0.491	0.017	SNAP sural nerve	-0.467	0.025

*CMAP* Compound motor action potential, *DML* Distal motor latency, *NCV* Nerve conduction velocity, *SNAP* Se&&nsory nerve action potential

## Discussion

In this study, we demonstrate that automated segmentation of peripheral nerve lesions using a convolutional neural network [7] is feasible and achieves robust performance across independent datasets. Importantly, segmented lesion burden correlates with electrophysiological parameters, characterizing nerve dysfunction and is so far regarded as the gold standard in the evaluation of peripheral nerves. Beyond feasibility, our results support this approach as a benchmark for standardized volumetric MRN biomarkers across cohorts.

MR neurography remains largely concentrated in specialized centers, in part because image interpretation and quantitative analysis require substantial expertise and manual effort. Automated delineation of peripheral nerves and intraneural lesions may lower this barrier by enabling standardized, reproducible volumetric quantification of lesion burden. Such automated quantification could facilitate large-scale studies, harmonized multicenter evaluation, and more objective assessment of structural nerve pathology.

### Automated lesion segmentation

**Fig. 2:**
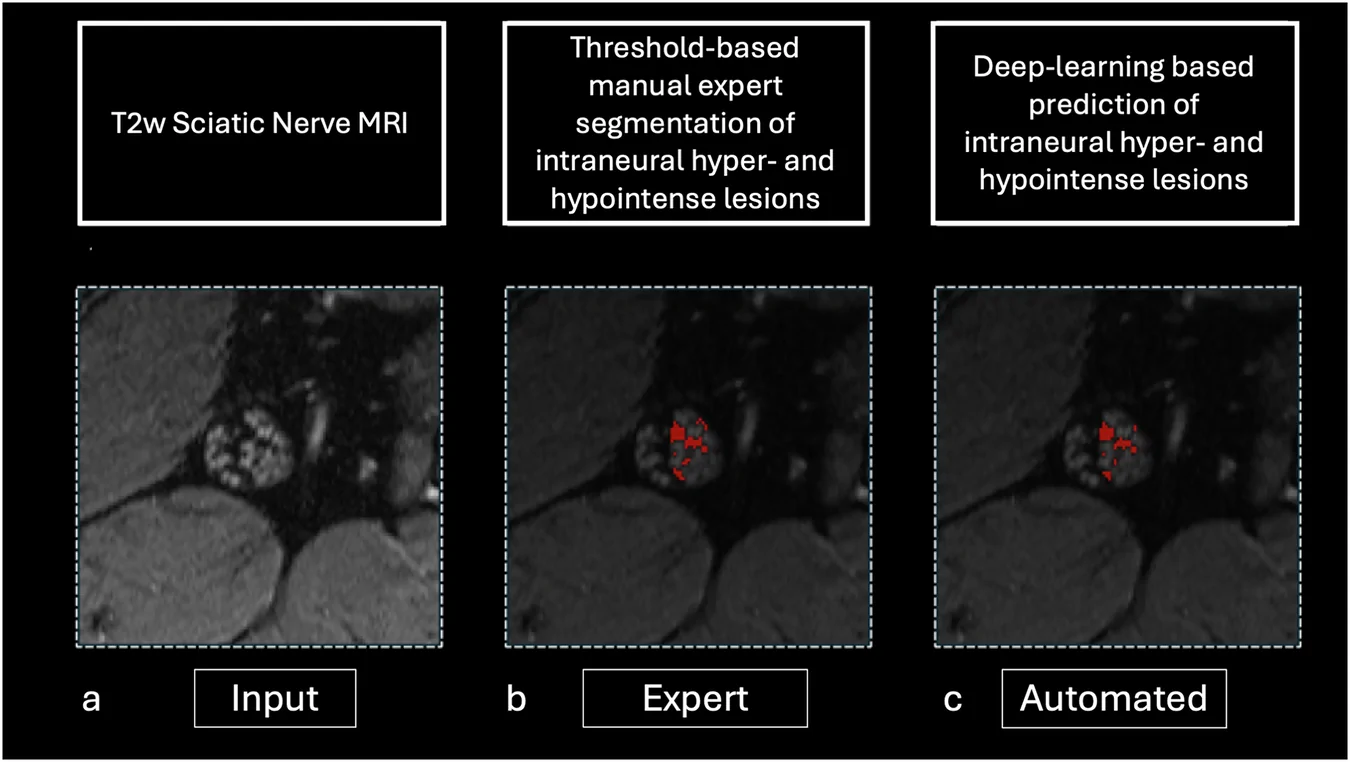
Representative lesion segmentation of the tibial division of the sciatic nerve. **a** Magnified T2w MRN of the sciatic nerve at the distal right thigh. **b** Manual, threshold-based segmentation of T2w hypointense lesions in the tibial division of the sciatic nerve. **c** Corresponding fully automated lesion segmentation using nnU-Net

Segmentation performance must be interpreted in light of known methodological constraints. Volumetric Dice scores are known to be highly size-dependent and disproportionately penalize minor boundary deviations in small structures [13, 14]. Accordingly, segmentation performance for small lesions is generally lower than for large space-occupying tumors or large peripheral nerves, where Dice values frequently exceed 0.80–0.90 [9, 15]. In contrast, reported Dice ranges for small lesion tasks such as multiple sclerosis lesions or small infarct region segmentation often lie between 0.50 and 0.70 ([16–19]). In this context, our achieved median Dice values of approximately 0.65–0.73 and surface Dice values around 0.70–0.77 indicate robust performance for small intraneural lesions. Surface-based metrics have been proposed as more size-invariant alternatives, particularly when evaluating small or irregular structures [20]. Interestingly, lesion segmentation performance was slightly higher when manually generated nerve masks were used compared with the fully automated pipeline. This difference was largely attributable to suboptimal segmentation of the tibial division of the sciatic nerve in a limited number of cases (Fig. 2). Inaccuracies in nerve boundary delineation propagated into lesion masks and reduced spatial overlap with the ground truth. The observed segmentation failures were predominantly attributable to anatomical and image-contrast–related challenges. In particular, vessels in proximity to the nerve introduced signal ambiguity and boundary confusion. Moreover, in anatomical regions with minimal interposing subcutaneous fat between the nerve and adjacent musculature, reduced tissue contrast impaired clear discrimination of nerve from muscle. These limitations are consistent with known challenges in small-structure segmentation and boundary-sensitive evaluation [13].

### Partial correlations to electrophysiological parameters

However, these localized technical inaccuracies did not translate into weaker biological associations. This dissociation between marginal differences in Dice performance and improved structure–function correlations in the fully automated pipeline underscores that traditional overlap metrics do not fully capture the structure-function relationship of quantitative imaging approaches. Partial correlation analyses in Test Set 2 showed equally strong and partly even better associations between lesion burden and electrophysiological impairment when lesion quantification was derived from fully automated nerve and lesion segmentation. The automated model showed significant negative correlations between hyperintense, hypointense, and overall lesion load and peroneal and tibial nerve conduction velocity and compound muscle action potential. These associations are physiologically plausible in neuropathies characterized by structural axonal damage or demyelination [4], where increased lesion burden would be expected to correspond to impaired conduction properties. The observation that structure-function relationships were comparable between the fully automated and manual workflows suggests that automated nerve segmentation does not compromise the biological validity of downstream quantitative measurements. Standardized algorithmic boundary definition may provide a level of consistency similar to expert-derived segmentations while reducing observer-dependent variability. Although minor differences in nerve contour placement can influence lesion-to-nerve volume ratios, these variations did not substantially alter the detected associations with electrophysiological parameters, supporting the robustness of the automated approach. By enforcing consistent geometric definitions across subjects, automated segmentation may improve reproducibility and enhance sensitivity to subtle disease-related structural alterations. Although the relatively small sample size of Test Set 2 (*n* = 31) necessitates cautious interpretation, the directionality and physiological plausibility of the observed associations support the robustness of this finding. The results therefore indicate that automated MRN-derived quantitative metrics showed associations with electrophysiological parameters at least comparable to those derived from manual quantification. These exploratory findings, while physiologically plausible, should be interpreted with caution given the limited sample size (*n* = 31), the absence of multiple comparisons correction, and the cross-sectional retrospective study design.

The discrepancy between relatively high PPV values and comparatively low sensitivity in the fully automated pipeline warrants comment. In Test Set 1 with expert nerve mask, median PPV was 0.949 (95% CI 0.927–0.981) for hyperintense and 1.000 (0.994–1.000) for hypointense lesions, while median sensitivity was 0.601 (0.502–0.669) and 0.522 (0.443–0.654), respectively. In the fully automated pipeline, median PPV remained high at 0.892 (0.852–0.935) and 0.805 (0.734–0.883), while sensitivity was 0.489 (0.407–0.608) and 0.394 (0.330–0.455). This conservative detection pattern means the model rarely generates spurious lesion findings but misses a proportion of true lesions. Clinically, this is relevant for individual-level longitudinal monitoring where underestimation of lesion burden may be meaningful, whereas at cohort level the high PPV supports reliability of volumetric metrics for group comparisons. Future work should evaluate whether adjusted post-processing thresholds or ensemble approaches can improve sensitivity without sacrificing PPV.

### Potential for clinical workflow

Automated segmentation and quantification of peripheral nerve lesions offers several opportunities for both clinical practice and research workflows. While MRN examinations remain concentrated in specialized centers due to the limited number of experienced readers, structured reporting systems such as the MRI-based Neuropathy Score Reporting and Data System aim to standardize neuropathy assessment through qualitative, morphology-based criteria [3]. Artificial intelligence-based quantitative assessment could complement such structured frameworks by enabling reproducible lesion burden measurements. Quantitative characterization of lesion distribution and burden has previously provided novel insights into structural lesion patterns and their clinical relevance in diabetic neuropathy [4, 5, 21–25]. The ability to automatically quantify absolute lesion volume and lesion-to-nerve volume ratios represents an important step toward establishing imaging-based biomarkers in peripheral neuropathies. While MRN interpretation is currently largely qualitative and experience-dependent, volumetric quantification provides objective structural metrics. Such quantitative markers may serve as complementary disease severity indicators alongside electrophysiological testing and may facilitate harmonized imaging endpoints in multicenter trials. Although clinically relevant threshold values cannot yet be defined, the stable lesion-to-nerve ratios observed across independent datasets support the feasibility of future normative range development. It must be emphasized that the present study demonstrates technical feasibility and biological plausibility rather than immediate clinical applicability. Translation into clinical practice will require prospective validation, multicenter reproducibility studies and demonstration of diagnostic or prognostic added value over existing measures.

### Limitations

Several limitations must be acknowledged. First, this was a retrospective study and therefore subject to potential selection bias. In addition, the training cohort predominantly consisted of patients with type 2 diabetic neuropathy, which may limit disease-specific generalizability. However, external validation in Test Set 1, which included healthy controls as well as patients with neoplastic and neuropathic nerve lesions of non-diabetic origin, demonstrated stable segmentation performance across heterogeneous pathological conditions. This supports a certain degree of disease-independent robustness of the model, although further validation in larger, disease-specific cohorts remains warranted. Second, although multiple scanners were included, all examinations were performed at 3.0 T using similar institutional protocols. The robustness of the model across different field strengths, vendors, or non-standardized acquisition protocols remains to be determined. This is a clinically relevant limitation: in real-world deployment, MRN examinations may be acquired at different field strengths, with different receiver coil configurations or using non-standardized fat-suppression sequences such as STIR instead of chemical-shift fat saturation, all of which may alter lesion conspicuity and signal characteristics in ways not represented in the training data. Prospective multicenter studies covering diverse scanner platforms, field strengths, and acquisition protocols will be required before the framework can be deployed outside specialized MRN centers. Third, lesion segmentation was based on slice-wise thresholding after conversion of images to 8-bit format. While this approach ensured consistent and standardized annotations, the reduced grayscale range may have limited subtle signal differences and slightly influenced lesion boundary definition. This potential effect should be considered when interpreting segmentation performance. A further limitation concerns the potential overlap between intraneural vessels and T2w hyperintense nerve lesions. Although clearly identifiable linear or tubular vascular structures were excluded during manual mask correction whenever possible, small intraneural vessels could not be reliably distinguished from hyperintense intraneural lesions using the present threshold-based workflow. Therefore, rare inclusion of intraneural vessels within the T2w hyperintense lesion masks cannot be excluded. However, given their expected low frequency and limited spatial extent, this is unlikely to have substantially confounded the analysis of hyperintense lesion burden. Finally, the relatively small sample size of Test Set 2 limits statistical power for correlation analyses and increases susceptibility to type I or type II error. Multiple structure-function associations were explored, and findings should therefore be interpreted as hypothesis-generating.

## Conclusion

This study demonstrates that fully automated segmentation and quantification of T2w intraneural lesions in MR neurography is feasible and robust across independent datasets. Beyond technical performance, automated quantification showed biologically plausible correlations with electrophysiological impairment compared with manually derived measures, underscoring its potential as an objective imaging biomarker. While further prospective validation is required, automated MRN analysis represents a base toward standardized, quantitative assessment of peripheral nerve pathology. Taken together, the presented framework provides a reproducible reference for future multicenter and longitudinal MRN studies.

## Data Availability

The datasets generated and/or analyzed during the current study are not publicly available but are available from the corresponding author on reasonable request.

## Abbreviations

CNN: Convolutional neural networks
ICC: Intraclass correlation coefficient
MRI: Magnetic resonance imaging
MRN: Magnetic resonance neurography
PPV: Positive predictive value
T2w: T2-weighted
